# Pre‐school neurocognitive and functional outcomes after liver transplant in children with early onset urea cycle disorders, maple syrup urine disease, and propionic acidemia: An inception cohort matched‐comparison study

**DOI:** 10.1002/jmd2.12095

**Published:** 2020-01-27

**Authors:** Shailly Jain‐Ghai, Ari R. Joffe, Gwen Y. Bond, Komudi Siriwardena, Alicia Chan, Jason Y. K. Yap, Morteza Hajihosseini, Irina A. Dinu, Bryan V. Acton, Charlene M. T. Robertson, the Western Canadian Complex Pediatric Therapies Follow‐up Group

**Affiliations:** ^1^ Department of Medical Genetics University of Alberta Edmonton Alberta Canada; ^2^ Department of Pediatrics University of Alberta Edmonton Alberta Canada; ^3^ Department of Pediatrics Glenrose Rehabilitation Hospital Edmonton Alberta Canada; ^4^ School of Public Health (Biostatistics) University of Alberta Edmonton Alberta Canada; ^5^ University of Melbourne The Royal Children's Hospital Melbourne Australia; ^6^ Department of Psychology University of Saskatchewan Saskatoon Saskatchewan Canada

**Keywords:** functional outcomes, inborn errors of metabolism, liver transplantation, neurocognitive outcomes, pre‐school, urea cycle disorder

## Abstract

**Background:**

Urea cycle disorders (UCD) and organic acid disorders classically present in the neonatal period. In those who survive, developmental delay is common with continued risk of regression. Liver transplantation improves the biochemical abnormality and patient survival is good. We report the neurocognitive and functional outcomes post‐transplant for nine UCD, three maple syrup urine disease, and one propionic acidemia patient.

**Methods:**

Thirteen inborn errors of metabolism (IEM) patients were individually one‐to‐two matched to 26 non‐IEM patients. All patients received liver transplant. Wilcoxon rank sum test was used to compare full‐scale intelligence‐quotient (FSIQ) and Adaptive Behavior Assessment System‐II General Adaptive Composite (GAC) at age 4.5 years. Dichotomous outcomes were reported as percentages.

**Results:**

FSIQ and GAC median [IQR] was 75 [54, 82.5] and 62.0 [47.5, 83] in IEM compared with 94.5 [79.8, 103.5] and 88.0 [74.3, 97.5] in matched patients (*P*‐value <.001), respectively. Of IEM patients, 6 (46%) had intellectual disability (FSIQ and GAC <70), 5 (39%) had autism spectrum disorder, and 1/13 (8%) had cerebral palsy, compared to 1/26 (4%), 0, 0, and 0% of matched patients, respectively. In the subgroup of nine with UCDs, FSIQ (64[54, 79]), and GAC (56[45, 75]) were lower than matched patients (100.5 [98.5, 101] and 95 [86.5, 99.5]), *P* = .005 and .003, respectively.

**Conclusion:**

This study evaluated FSIQ and GAC at age 4.5 years through a case‐comparison between IEM and matched non‐IEM patients post‐liver transplantation. The neurocognitive and functional outcomes remained poor in IEM patients, particularly in UCD. This information should be included when counselling parents regarding post‐transplant outcome.

**Abbreviations**
ABAS‐II: Adaptive Behavior Assessment System, Second Edition
ADHD: attention deficit hyperactivity disorder
ADOS: Autism Diagnostic Observation Schedule
ASD: autism spectrum disorder
FSIQ: full‐scale intelligence quotient
GAC: Adaptive Behavior Assessment System II‐general adaptive composite
IEM: inborn errors of metabolism
IQR: interquartile range
LT: liver transplant
MSUD: maple syrup urine disease
OAD: organic acid disorder
PA: propionic acidemia
SD: standard deviation
UCD: urea cycle disorder
VABS: Vineland Adaptive Behaviour Scale
WPPSI‐III: Wechsler Preschool and Primary Scale of Intelligence, Third Edition

## 1 INTRODUCTION

Inborn errors of protein metabolism are inherited disorders due to single enzyme or cofactor deficiency leading to accumulation of toxic metabolites and deficiency of substrates. Urea cycle disorders (UCD), maple syrup urine disease (MSUD), and propionic acidemia (PA) fall under this classification and clinically present in the neonatal period with feeding intolerance, seizures, and encephalopathy progressing to coma. Neonatal onset (≤30 days) of these inborn errors of metabolism (IEM) is associated with a poor prognosis for survival and neurological outcomes.^1^ Those who survive the initial presentation remain at risk of ongoing metabolic decompensations including hyperammonemia, hyperleucinosis, and metabolic acidosis. The cumulative effects of these recurrent decompensations are thought to lead to global developmental delay with high risk of regression of acquired skills and death. In addition to intellectual disability and abnormal brain imaging,^2^, ^3^ individuals with UCD are at risk of cerebral palsy, seizures, and cortical blindness.^4^ Individuals with PA can also have significant intellectual disability and additionally show higher prevalence of autism spectrum disorder (ASD), optic nerve atrophy, and basal ganglia strokes.^5^ Intellectual disability in MSUD is present,^6^ although not as severe as in UCD and PA. In MSUD, attention deficit, hyperactivity, and mental health illnesses are prevalent.^7^

Liver transplantation (LT) in these disorders has been shown to correct or significantly improve the biochemical abnormalities. In UCDs, a successful LT eliminates hyperammonemic crises, need for dietary protein restriction and ammonia scavenger therapy. In PA and MSUD, the biochemical abnormalities are significantly attenuated after LT, although some degree of dietary, medical, and illness management is still indicated as there remains a risk of metabolic decompensation.^8^, ^9^ Complications following LT include thrombosis, infection, post‐transplant lymphoproliferative disease, multiorgan failure, graft loss, and death.^10^ However, the literature is describing improved medical outcomes and survival in children,^10^, ^11^, ^12^, ^13^ possibly due to fewer pre‐LT chronic liver disease comorbidities, and as such frequency of LT for UCDs, MSUD, and organic acid disorders (OAD) has increased in the last 10 years.^10^

Recent studies have also described developmental and cognitive performances after LT for IEM. The United Network for Organ Sharing (UNOS) database reported that in 323 pediatric IEM patients approximately 54 months following LT, 40% of UCD, 79% of OAD, and 22% of MSUD patients had cognitive delays.^10^ In that study, objective measures were not used as data were collected through questionnaires. Data from the urea cycle consortium presented neuropsychological assessment on 528 individuals with 8 different enzyme/transporter defects; however, patients were assessed at different ages, the majority had late onset phenotype, and LT status was unknown.^14^ Significant variability dependent on age and specific UCD was noted, highlighting difficulties in comparing neonatal onset with late onset phenotype.^14^ Stevenson et al found 3/7 (43%) school aged children with UCD post‐LT to be 1 to 2 SD (15‐30 points) below population norms in intelligence quotient.^15^ In an OTC series of four patients, post‐LT full scale intelligence quotients (FSIQ) were 75, 73, 68, and 49.^16^ At best, LT may halt further neurological insult but does not reverse underlying injury.^10^, ^17^, ^18^, ^19^ In comparison, a study in non‐IEM pediatric patients having had LT at age <3 years demonstrated FSIQ on average to be within half a SD of population norms.^20^ This suggests that IEM patients with LT, while have fewer complications than those with LT for other reasons,^11^ likely have worse cognitive outcomes post‐LT.

The pre‐LT neurological injury and other comorbidities as well as ongoing biochemical abnormalities likely play an important role and need a detailed description to allow better understanding of the post‐LT outcome. As each center has limited number of patients, the reported literature lacks concordance of the phenotype, with varying severity of same disease included in the same cohort.^14^, ^16^, ^17^ Given access to resources, and variability in IEM and LT care between centers, appropriate comparisons are difficult to draw. In this study, we hoped to address some of these gaps by comparing outcomes at 4.5 years of age between 13 severe (presenting in the neonatal period) IEM patients and 26 matched non‐IEM post‐LT patients at our Western Canadian referral center. IEM patients rarely have liver dysfunction pre‐LT, while non‐IEM patients usually do [e.g., hypoalbuminemia, ascites, hepatic encephalopathy]; and IEM patients often have metabolic crises pre‐LT, while non‐IEM patients rarely do. Therefore, we hypothesized that neurological outcomes will be worse for IEM patients while growth and health outcomes will be similar to non‐IEM patients post‐LT, and that some pre‐LT variables may be associated with adverse outcomes.

## 2 METHODS

### 2.1 Study description

Through the prospective, longitudinal, interprovincial inception cohort study, the Western Canadian Complex Paediatric Therapies Follow‐up Program (CPTFP), children from western Canada and corresponding northern territories who have complex therapies in Alberta, Canada receive neurodevelopmental follow‐up. All patients in this study had LT at the Stollery Children's Hospital, Edmonton, Canada. For children transplanted before their sixth birthday, referral for follow‐up was made by the attending hepatologist at the time of the liver transplant. Details of the registration procedure for each child have been previously described.^21^ When survival is deemed likely, a nurse coordinator registers the child and discusses follow‐up procedures with the parents. Parents understand the dual purpose of follow‐up to include service for possible developmental concerns for their child and parental psychosocial support as well as an audit of outcome and research. Contact is made with the developmental follow‐up clinic at the tertiary site of referral.

From 2000 to 2016, LT was performed in nine patients with UCD, one with PA, and three with MSUD. The patients with UCD included three with carbamylphosphate synthase‐1 deficiency (CPS1‐D), three with ornithine transcarbamylase deficiency (OTC‐D; all males), one with argininosuccinate synthase deficiency (Citrullinemia type 1) and 2 with argininosuccinate lyase deficiency. Case‐comparison matching was completed using the following variables: year of transplant (within 2 years), sex, gestational age (within 2 weeks), age at transplant (within 6 months), and socioeconomic status (SES) based on employment of the main wage earner in the household (population mean 43 and SD 13, matched within 15 points).^22^ Each IEM patient was matched individually with two other non‐IEM children on each of these variables from the established CPTFP database of prospectively collected acute care and outcome data. Indications for LT for the non‐IEM children included biliary atresia, acute liver failure, cholestasis, and tumor.

Acute care variables from the established database included: gestational age; sex; time on waiting list; age, weight, height, creatinine, West Haven Classification of encephalopathy at LT,^23^ postoperative LT days on ventilation, intensive care and hospitalisation, reoperation within 30 days, and retransplant within 1 year. Data on encephalopathy at LT obtained from the established database are characterized based on symptoms at the time of transplantation only. Dependent on biochemical parameters, IEM patients likely experienced encephalopathy at diagnosis and around metabolic decompensations. A retrospective chart review was performed for all patients and additional data were collected on initial presentation, ammonia at presentation, number of hyperammonemic crises (defined as ammonia over 100 μmol/L) pre‐ and up to 2 years post‐LT and use of renal replacement therapy. To better understand the neurological profile pre‐LT, clinical description of development as described in the chart and when available, Vineland Adaptive Behavior Scale (VABS) outcomes were recorded. Seizures, abnormal neurological findings (defined as any of seizure, hypotonia, stroke‐like episode, or encephalopathy at LT) and brain imaging data were also collected. For the IEM patients, metabolic treatment including dietary management and protein restriction, IEM medications, and peri‐LT management was recorded.

### 2.2 Assessment and measures

Through the CPTFP, all children at their respective referral sites have multidisciplinary assessments performed post‐LT; pre‐school (4‐6 year) outcomes are reported here. Neurological examinations are completed by a neurodevelopmental pediatrician or pediatrician experienced in developmental follow‐up; diagnoses of motor disability are confirmed by a neurologist; and visual impairment, defined as corrected visual acuity in the better eye of <20/60, by an ophthalmologist; growth measurements are completed. A detailed health history is recorded. Experienced paediatric psychologists assess the neurocognitive ability using the gold‐standard Wechsler Preschool and Primary Scales of Intelligence (third edition)^24^ to give a FSIQ with normative US population mean and SD of 100 (15). Pediatric‐experienced audiologists assess bilateral hearing in a sound‐proof booth; sensorineural hearing impairment is defined as responses in the better ear of >25 dB at any frequency from 250 to 4000 Hz. Functional outcomes are determined using a parent‐completed questionnaire, the Adaptive Behavior Assessment System II (ABAS‐II).^25^ The ABAS‐II evaluates realistic, independent behaviors of patients, the effectiveness of interaction with others, within community contexts. The measure includes four domains: conceptual (communication, functional pre‐academics, and self‐direction), practical (home living, health and safety, community use, and self‐care), social (leisure and social), and overall general adaptive composite (GAC), which includes all of the above as well as motor skills. The composite GAC age‐based population score has a mean (SD) of 100 (15). In addition some children's parents were given a similar questionnaire pretransplant, the VABS with an overall composite score mean (SD) of 100 (15).^26^ ASD was prospectively diagnosed using the gold standard DSM‐IV‐TR prior to 2014 and thereafter DSM‐5 criteria^27^, ^28^ by multidisciplinary teams at each site with supplement from the appropriate module of the Autism Diagnostic Observation Schedule (ADOS) and parental interviews and observations.

### 2.3 Perioperative management of IEM patients

All IEM patients received intravenous dextrose 10% (glucose infusion rate of 6‐11 mg/kg/min) with appropriate electrolytes and intravenous 20% lipids (1.5‐3 g/kg/day) immediately prior to and during the transplant procedure. All UCD patients received continuous infusion of IV ammonia scavengers (sodium phenylacetate and sodium benzoate, Ammonul) and IV Arginine during the operation. Information was not available on one patient. Ammonia monitoring was requested but not performed in the majority of cases in the operating room. Perioperative or intraoperative dialysis was not performed in any patient. Immediately after transplant, UCD patients were started on typical post‐LT parenteral nutrition without any protein restriction. PA and MSUD patients were started on protein restricted diets and in most, protein was started at half daily requirements and increased to 1.5 to 2 g/kg/day by day 7 post‐LT, based on biochemical parameters including acid base status and plasma amino acids. These patients have sick day and metabolic emergency plans post‐LT, which is not the case for UCDs. The goal of sick day and emergency plans is to achieve lower protein, higher calories, and higher fluid intake.

### 2.4 Ethics

This study has been approved by the local health research ethics boards from all sites. All parents/guardians signed informed consent.

### 2.5 Statistical analysis

Variables are described as mean (SD), median (interquartile range [IQR]), or count (percentage) as appropriate. The first objective was to compare outcomes in metabolic and matched patients; Wilcoxon rank‐sum test was used to compare continuous outcomes. For dichotomous outcomes, a conditional logistic regression with an exact option was needed to address the sparse data in the matched pairs design. The algorithm did not converge, so that the data are described using counts and percentages, without statistical significance testing. The second objective was to compare outcomes of FSIQ and GAC in UCD and matched patients using the Wilcoxon rank‐sum test. The third objective was to compare variables between UCD and individually matched patients, in order to assess what may account for any differences found from objective two. Variables were compared using Wilcoxon rank‐sum test for continuous variables, and as above, no statistical testing was done for dichotomous variables. A *P*‐value of ≤.05 was used to determine statistical significance. A similar analysis was not performed for MSUD and PA as numbers were too small to allow for statistical end points to be met.

## 3 RESULTS

### 3.1 General description of the IEM patients

Table 1 summarizes the clinical presentation of the 13 patients with IEM. Age at transplant was 18.8 (14.1) months in IEM patients, and 17.6 (14.7) months in non‐IEM patients (Wilcoxon rank‐sum test *P* value = .81). The time on waitlist was 93.8 (65.6) days in IEM patients, and 65.6 (72.5) days in non‐IEM patients (*P* = .25). Of UCD patients, 8/9 (89%) had severe neonatal phenotype based on presentation at <5 days of life with ammonia >500 μmol/L (one patient presented at 30 days of life). All MSUD patients were diagnosed at <10 days of life with leucine >1500 μmol/L and as such are classified as severe neonatal phenotype. The PA patient was diagnosed through newborn screening but was symptomatic with lethargy, hyperammonemia, elevated anion gap, and mild lactic acidosis, thus considered to be neonatal onset. Of the IEM patients 7/13 (54%) received dialysis at diagnosis, including six with UCD. Given patients were transferred from other centers, pre‐LT hyperammonemia events are not available on all patients and the majority of recorded events are those that took place in our province; however, it is unlikely that a severe crisis (ammonia >500 umol/L) was missed during chart review even from other provinces as this would have resulted in hospital admission. Of UCD patients, 7/9 (78%) had multiple mild hyperammonemic crises (100‐199 μmol/L), and all had at least one severe hyperammonemic crisis (>500 μmol/L; Table 1), while none of the matched patients had any pre‐LT hyperammonemic events. Dietary protein restriction was used for all IEM patients pre‐LT. Twelve IEM patients had brain MRI pre‐LT with 10 (83%) reported as abnormal. Abnormalities on MRI included signal abnormality in white matter or brainstem with variable degrees of edema, gliosis, and atrophy; two patients had intracerebral hemorrhages on MRI of the brain.

**Table 1:** Descriptive information for the inborn errors of metabolism patients

IEM	Age at dx (days)	Ammonia at dx (μmol/L)	Pre‐LT hyperammonemic events (number)					Motor exam pre‐LT^a^	Delay noted on chart pre‐LT (age)^a^	Age at LT (mos)	Pre‐LT protein restriction (g/kg/day)	Post‐LT protein restrict‐ion
			100‐199 μmol/L	200‐299 μmol/L	300‐499 μmol/L	500‐999 μmol/L	≥1000 μmol/L					
UCD	<5	750‐999	12	4	2	1	0	Hypotonia, Seizure	VABS 85 (5 mo) “Motor delay”	6‐12	1.2 (50%‐70%N)	No
UCD	<5	999‐1500	70	27	7	1	1	Hypertonia, Choreiform, Seizure	“Severe GDD”	12‐24	1.0 (50% N)	No
UCD	<5	>1500	N/A	N/A	N/A	N/A	1	Hypotonia, Hypertonia, Seizure	VABS 94 (4 mo)	6‐12	1.0	No
UCD	<5	500‐750	20	7	1	1	0	Febrile Seizures	“Motor delay” “Receptive and expressive language delay” (33 mo)	24‐48	1.4 (35% N)	No
UCD	<5	999‐1500	2	1	0	1	2	‐	‐	6‐12	1.5 (20% N)	No
UCD	<5	750‐999	13	1	0	2	0	Hypotonia	“Motor delay”	6‐12	1.2 (20% N)	No
UCD	6‐30	500‐750	4	N/A	N/A	1	0	Seizure	“Mild delay” (18 mo)	12‐24	0.8 (20% N)	No
UCD	<5	>1500	37	17	5	0	5	Seizure	VABS 76 (3 mo)	<6	1.5‐2.5 (50% N)	No
UCD	<5	999‐1500	N/A	N/A	N/A	N/A	1	Hypertonia	“Normal” (19 mo)	12‐24	1 (all N)	No
PA	<10	150‐200	0	0	0	0	0	‐	“GDD”, VABS 70 (12 mo)	12‐24 m	1.6 (60% N)	DRI
		Leucine at dx (μmol/L)									Pre‐LT leucine restriction (mg/day)	
MSUD	<10	>1500						Hypertonia	“VABS Average” (2.5 years)	2‐4 Years	Leucine 200‐400	No
MSUD	<10	>1500						Hypertonia	“Normal” (2.5 years)	2–4 Years	Leucine 200‐400	No
MSUD	<10	>1500						‐	“Motor delay” VABS 85 (10mos)	12‐24 mo	Leucine 200‐400	No

Abbreviations: DRI, daily reference intake; Dx, diagnosis; GDD, global developmental delay; LT, liver transplantation; MSUD, maple syrup urine disease; N, natural protein; N/A, not available from chart review; OAD, organic acid disorder; UCD, urea cycle disorder; VABS, Vineland Adaptive Behavior Scale.

a Motor exam and delay noted in chart is as described in the clinical chart of the patient pre‐LT.

### 3.2 Post‐LT metabolic course in IEM patients

No hyperammonemic episodes post‐LT are reported in the UCD patients at 4.5 years of age and all remain on typical diet with no protein restriction. All OTC‐D and CPS1‐D patients remain on citrulline post‐transplant due to low citrulline values on plasma amino acid analysis. By day 30, MSUD and PA patients were above 2 g/kg/day of natural protein and only one patient remained on a small amount of metabolic formula. MSUD patients are not on protein restriction outside of illness. Elevations in leucine have been observed with illness in MSUD patients post‐LT and in one patient this led to neurological symptoms. PA patient remains on protein restriction to meet daily requirement and remains on carnitine. Hyperammonemic episodes have not been observed in the PA patient post‐LT even with illness although illness management was used with need for hospitalization.

### 3.3 Outcomes at 4.5 years of IEM (UCD, MSUD, and PA) patients

**Figure 1:**
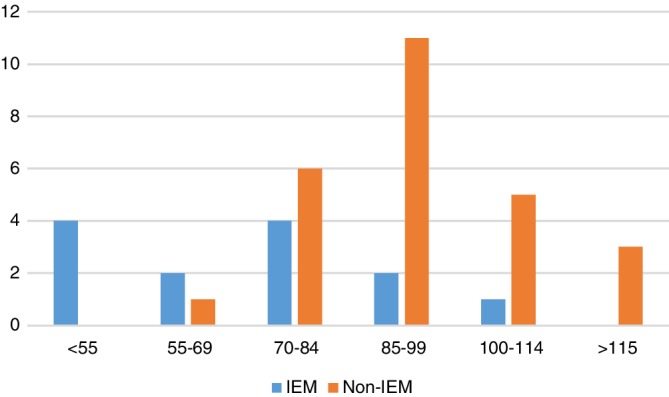
Full scale intelligence quotient for IEM and non‐IEM patients. The figure outlines the distribution of FSIQ for the IEM and non‐IEM patients. The X‐axis has FSIQ categories (for each population norm SD of 15 interval) and the Y‐axis indicates the number of patients in each category. For the general population, FSIQ score mean (SD) is 100 (15). Scores <70 occur in 2.27% of the normative population. IEM, inborn errors of metabolism, FSIQ, full scale intelligence quotient

Outcomes between IEM and matched comparison patients are given in Figure 1 and Table 2. Medical outcomes were similar, including weight and height *z*‐scores, number of hospitalizations, medications, and specialists involved in care. Five (39%) of IEM patients compared with 0% from the comparison group had a gastrostomy tube at 4.5 years of age. All patients survived and completed assessments at 4.5 years of age. Children with IEMs had statistically significantly lower FSIQ and GAC at 4.5 years of age compared with non‐IEM patients; 7 (54%) and 7 (54%) had FSIQ and GAC <70 compared with 1 (4%) and 3 (12%) of non‐IEM patients, respectively (Table 2). In the IEM patients, four had FSIQ <55, three were between 55 and 69, three were between 70 and 84, two were between 85 and 99, and one was over 100. In the IEM patients, ASD was diagnosed in 5 (39%) (4 UCD, 1 PA) and cerebral palsy in 1 (8%; UCD), whereas these diagnoses were absent in the non‐IEM comparison group. All children with a diagnosis of ASD had a FSIQ and GAC <70 and all met the diagnosis of ASD using both the DSM criteria and after the ADOS was completed. Hearing loss was present in 1(8%) and 2 (8%) patients in the IEM and non‐IEM groups, respectively. In the non‐IEM group, the hearing loss is a complication of a chemotherapy agent. Of note, none of the three MSUD patients had FSIQ <70, intellectual disability (FSIQ and GAC <70), or ASD.

**Table 2:** Comparison of outcomes in inborn errors of metabolism and matched comparison patients

Outcome	IEM patients (n = 13) Median [IQR], n (%)	Comparison patients (n = 26) Median [IQR], n (%)	*P*‐value
*Neurocognitive, functional, and neurological outcomes*			
FSIQ	75.0 [54.0, 82.5]	94.5 [79.8, 103.5]	<.001
FSIQ <70	7 (54%)	1 (4%)	‐
GAC	62.0 [47.5, 83.0]	88.0 [74.3, 97.5]	.003
GAC <70	7 (54%)	3 (12%)	‐
Intellectual disability [both FSIQ and GAC <70]	6 (46%)	0 (0%)	‐
Cerebral Palsy	1 (8%)	0 (0%)	‐
Hearing loss	1 (8%)	2 (8%)	‐
Autism spectrum disorder	5 (39%)	0 (0%)	‐
Seizures	1 (8%)	0 (0%)	‐
*General medical outcomes post‐LT*			
Weight z‐score	−0.2 [−2.1, 0.9]	−0.9 [−1.6, 1.0]	.48
Height z‐score	−0.3 [−0.7, 1.0]	0.4 [−0.6, 1.0]	.65
Gastrostomy tube	5 (39%)	0 (0%)	‐
Number of hospitalizations for liver problems	0 [0, 1]	0 [0, 2]	.55
Number of hospitalizations not for liver problems	1 [0, 2]	1 [0, 2.25]	.94
Number of specialists involved in care	2 [1, 3]	1.5 [1, 3]	.77
Number of medications	3 [1, 4.5]	2.5 [1, 5]	.98

Abbreviations: FSIQ, full scale intelligence quotient; GAC, general adaptive composite; IEM, inborn error of metabolism; IQR, Interquartile range.

### 3.4 Outcomes at 4.5 years in the UCD only and matched comparison children

The UCD patients differed in some respects from their matched cohort (Table 3). Most significantly, in UCD patients the FSIQ of 64 [IQR 54, 79] and GAC of 56 [45, 75] were much lower than in the matched patients (*P* = .005 and .003 respectively, Table 3). The UCD patients also had higher ammonia at diagnosis (median 1100 umol/L) and many more episodes of hyperammonemia (median 19, [IQR 3.5, 85]) than the matched cohort (median 42 umol/L and median 0, [0, 0] events), *P* < .001. ASD was diagnosed in 4 (44%) UCD patients. Abnormal neurological examination and seizures preoperatively were also more common in the UCD patients (Table 3).

**Table 3:** Comparison of urea cycle disorder and matched comparison patients

Variable	UCD (n = 9) Median [IQR], n (%)	Matched comparison (n = 18) Median [IQR], n (%)	*P*‐value
*Pre‐LT variables*			
Birth gestational age (weeks)	40 [40, 40]	40 [38, 40]	.44
Sex (male)	9 (100%)	18 (100%)	‐
Weight (kg)	9.8 [8.3, 12.1]	7.8 [7, 9.8]	.095
Weight *z*‐score	0.3 [−1.1, 0.9]	−1.2 [−1.7, −0.4]	.034
Height (cm)	67.0 [60.8, 80.7]	68.5 [65, 78.7]	.54
Height z‐score	−1.7 [−1.9, −0.06]	−1.5 [−2, 0.02]	.92
Waitlist days	97.0 [7.5, 138.5]	45.0 [16.7, 66.7]	.37
Age at LT (months)	10.5 [7, 19]	11.0 [7, 18.7]	.90
Hospital days	30.0 [5.5, 60]	11.0 [1.7, 45]	.39
PICU days	1 [0, 22]	0 [0, 0]	.010
Ammonia at diagnosis μmol/L	1100 [723.5, 1484.5]	42 [36, 61]	<.001
Episodes of ammonia >100 μmol/L	19.0 [3.5, 85]	0 [0, 0]	<.001
Creatinine (μmol/L)	25.0 [11, 84.5]	17.5 [11.5, 25.7]	.27
Renal replacement therapy	6 (67%)	0 (0%)	‐
Encephalopathy at LT	1 (11%)	2 (11%)	‐
Abnormal neurological examination	8 (89%)	1 (6%)	‐
Seizures	6 (67%)	0 (0%)	‐
Abnormal neuroimaging	6/8 (75%)	1/16 (6%)	‐
*Post‐LT variables*			
Ventilation days	4.0 [2.5, 15]	3.0 [2, 7.2]	.30
PICU days	13.0 [4.5, 26.5]	6.5 [4, 17.5]	.42
Hospital days	44.0 [27.5, 87.5]	38.5 [23, 91.7]	.61
Number of days tacrolimus level >15 ng/mL (first 0‐30 days)	3 [2, 4]	3 [1, 5]	.92
Creatinine (μmol/L)	40.0 [23.5, 74]	32.5 [23.7, 40]	.25
Renal replacement therapy within 30 days	3 (33%)	0 (0%)	
Reoperation within 30 days	7 (78%)	7 (39%)	‐
Retransplant within 1 year	0 (0%)	1 (6%)	‐
Abnormal neuro‐imaging	5/5 (100%)	None had neuro‐imaging	‐
*Follow‐up variables*			
SES	36.0 [34, 61.5]	41.0 [34.7, 48.5]	.98
FSIQ	64.0 [54.0, 79.0]	100.5 [98.5, 101.0]	.005
GAC	56.0 [45.0, 75.0]	95.0 [86.5, 99.5]	.003
Autism spectrum disorder	4 (44%)	0 (0%)	‐

Abbreviations: FSIQ, full scale intelligence quotient; GAC, general adaptive composite; IQR, interquartile range; LT, liver transplantation; PICU, paediatric intensive care unit; SES, socioeconomic status; UCD, urea cycle disorder. Abnormal neurological examination includes any of seizure, hypotonia, stroke‐like episodes [none had this], or encephalopathy at LT, as recorded in the chart notes.

## 4 DISCUSSION

Neonatal presentation of UCD, MSUD, and OAD are associated with a poor prognosis for survival and/or neurological outcomes.^1^, ^29^, ^30^ Natural history studies show survival without LT in those who survive the neonatal period to be 66% to 91% at 1 year of age for different types of UCDs.^29^ Post‐LT survival is significantly improved and some studies show survival to be 100%.^15^, ^17^ Quality of life improves^31^ and risk of further metabolic crises is significantly decreased (and absent in UCDs) after LT. With respect to the neurocognitive outcomes without LT, Krivitzky et al^32^ reported FSIQ in 13 neonatal onset UCD patients to average 65.5 (half had FSIQ <70) when assessed between 3 and 16 years of age. With LT, initial studies continue to show unsatisfactory neurocognitive outcomes and highlight the need for more objective data. We present neurocognitive data on 13 IEM patients post‐LT.

All of our cohort of IEM patients had neonatal onset phenotypes, and 8/9 UCD patients had a severe hyperammonemic event in the first 5 days of life. Our cohort had a significantly lower FSIQ and GAC at 4.5 years of age compared with the matched non‐IEM post‐LT patients. Almost half (46%) of the IEM patients, and none of the matched non‐IEM patients, had intellectual disability. Prevalence of autism (5/13 (39%) of IEM patients, 0 of non‐IEM patients), and cerebral palsy (1 (8%) of IEM patients, 0 of non‐IEM patients) was also higher in the IEM population at 4.5 years of age. Similar to what has been reported previously,^10^ our data suggest that MSUD is associated with better cognitive outcomes post‐LT, as none in our cohort had intellectual disability or ASD. However, these patients exhibited clinical concerns of anxiety, ADHD, and language disorders indicating post‐LT functional assessment remains important for optimal patient care.

Pre‐existing neurological injury and severity of hyperammonemic episodes needs to be documented as these may play a major role in the final neurocognitive outcome.^1^, ^33^ More UCD patients had pre‐LT abnormal neurological examination, seizures, abnormal neuroimaging, and a concern about developmental delay documented on the hospital chart, than in the non‐IEM patients (Table 3). In addition, ammonia at diagnosis was high, and episodes of hyperammonemia were frequent in the UCD patients, and did not occur in the non‐IEM patients. Ammonia is a known neurotoxin and initial hyperammonemia is known to strongly influence subsequent intellectual development.^34^ Thus, repeated hyperammonemic crises pre‐LT and potentially other toxic compounds like high glutamine, high citrulline, and low arginine may further contribute to the poor UCD post‐LT outcomes.^14^ Arginine plays a role in nitric oxide and creatine synthesis^35^ and dysregulation of these pathways may further contribute to neurological injury in UCDs. In PA, mitochondrial function is impaired leading to abnormal energy metabolism and increase susceptibility to neurological injury.^5^ In MSUD, high leucine levels predispose to encephalopathy and neurological injury. Thus, pre‐LT events may account for much of the differences between IEM and non‐IEM patients both pre‐ and post‐LT in neurological and functional outcomes.

Although guidelines recommend early LT in UCD patients,^36^ data from UNOS did not find that patients having LT at <2 years of age did better cognitively compared to patients having LT after 2 year of age.^10^ Of our IEM patients, 10 (77%) had LT at <2 years of age and from these, 5 (38%) were transplanted at <1 year of age. Thus, it may not be age at LT but more so the severity and duration of metabolic decompensations that play a significant role in determining neurocognitive outcomes. The neonatal brain may also be more vulnerable to a severe hyperammonemic insult^3^ but the effect of this damage may not be apparent till the child is older. General predictors of post‐LT adverse neurocognitive outcomes are variable in the literature, but may include pre‐LT poor growth, malnutrition, encephalopathy at LT, post‐LT neurotoxic medications, and clinical instability (eg, inotropes and high serum creatinine).^20^ These predictors were similar between the IEM and non‐IEM patients in our study. However, two potential exceptions need to be highlighted. First, given the severe protein restriction in the IEM patients pre‐LT, it is likely that malnutrition is present but not reflected in weight and height measurements. Second, while encephalopathy was not different at time of transplant in the two groups, encephalopathy during any period prior to LT (especially at presentation) is assumed in the IEM cohort. As such, it is not yet clear that earlier LT in neonatal onset IEM phenotype could improve the poor neurocognitive outcomes we report. In addition, performing LT at younger ages is associated with more frequent postoperative complications and mortality and this would need to be balanced in any decisions made.^37^

There are important limitations in this study. First, the study has a small cohort of n = 13 IEM patients (with n = 9 UCD patients), and n = 26 matched non‐IEM patients, undergoing LT at a single referral center. Second, being observational in design, we cannot prove cause and effect relationships. We hypothesize that the poor outcomes in neonatal onset IEM are due to neurological insults inherent from the underlying disease in infancy preceding LT. We did not match for hyperammonemia episodes, as this was not possible given its rarity in non‐IEM patients, and we hypothesized it as a likely explanation of differences in outcomes. In addition, we do not know whether the outcomes in IEM patients would be different without LT, as the study design did not match to IEM patients not having LT. Third, some of the retrospectively collected variables on neurological findings, development, and neuro‐imaging are subject to reporting bias for recording in the charts. Ideally, we would have objective assessments on all subjects pre‐LT; however, even if this was available, the literature supports the inability to use objective formal assessments in infantile period to predict cognitive profile at school age.^32^ Fourth, the matched comparison group included patients with heterogeneous causes of liver disease, and it is possible that specific subgroups of non‐IEM patients may have similarly poor outcomes to the IEM group. Nevertheless, in our follow‐up program the outcomes of acute liver failure patients transplanted at age <3 years include FSIQ of 92,^20^ suggesting that the IEM group does more poorly than even this high‐risk subgroup of non‐IEM patients. Fifth, the small sample size precluded our testing for predictors of adverse outcome in the IEM cohort.

Our study addressed some deficiencies in the literature. Strengths of the study are the 100% long‐term 4.5 year follow‐up on all IEM and matched non‐IEM LT patients, and the detailed outcome assessments done using validated instruments on all patients. In addition, the IEM patients were a homogeneous cohort of neonatal onset phenotypes. Although objective assessments were not available on all patients pre‐LT, we were successful in outlining a general view of an IEM patient's developmental and neurological profile including brain imaging pre‐LT. This, in addition to highlighting the metabolic course with dietary therapy and hyperammonemic crises allows a better understanding of the complex medical issues faced by IEM patients pre‐LT. The finding that IEM, and UCD patients in particular, have poor neurocognitive outcomes and high incidence of ASD, even after early LT, are concerning and requires further study. It may be helpful to have neurocognitive outcomes in younger affected siblings who were diagnosed prenatally. They may have avoided the initial severe hyperammonemic insult due to the early diagnosis and as such, their neurocognitive outcomes may be different.

## 5 CONCLUSION

Neonatal forms of UCD, MSUD, and PA are at lifelong risks of metabolic decompensation and each episode carries a risk of sustained neurological injury and death. LT is primarily considered as a treatment option to eliminate or significantly decrease the ongoing risk of such metabolic decompensations and increase survival. Our data aligns with reported excellent patient survival along with elimination of hyperammonemic crisis in UCD patients and significantly decreased metabolic decompensations in MSUD and PA patients. The goal of our study was to evaluate FSIQ and GAC at age 4.5 years through a case and matched‐comparison between IEM and non‐IEM patients post‐LT. Our data shows in neonatal forms of UCDs, MSUD, and PA, the neurocognitive and functional outcomes remain poor. Particularly in UCDs, there were concerning FSIQ and GAC with high incidence of intellectual disability and ASD. Thus, it becomes crucial that families are appropriately counselled regarding the likelihood of poor neurological outcome even after a successful LT for neonatal IEM phenotypes.

## CONFLICT OF INTEREST

Shailly Jain‐Ghai has participated in advisory boards and received honoraria and travel grants from Sanofi‐Genzyme, Horizon Pharmaceutical, Amicus, BioMarin and Shire.

Ari R Joffe, Gwen Y Bond, Komudi Siriwardena, Alicia Chan, Jason Y K Yap, Morteza Hajihosseini, Irina A Dinu, Bryan V Acton and Charlene MT Robertson have no conflicts of interest to declare.

## AUTHOR CONTRIBUTIONS

S.J.‐G. data collection, interpretation, study design, drafting, and revision of manuscript. G.Y.B. data collection, revision of manuscript. I.A.D. and M.H. statistical analysis and revision of manuscript. K.S., A.C., B.V.A. revision of manuscript. J.Y.K.Y. study design and revision of manuscript. A.R.J. and C.R. study design, data interpretation, drafting and revision of the manuscript. All authors reviewed and approved the manuscript.

## ETHICS APPROVAL STATEMENT

The study was approved by the institutional review board, The Health Research Ethics Board, at the University of Alberta, Edmonton, AB, Canada. The decision number is Pro00001030.

## INFORMED CONSENT

All procedures followed were in accordance with the ethical standards of the responsible committee on human experimentation (institutional and national) and with the Helsinki Declaration of 1975, as revised in 2000. Informed consent was obtained from all patients for being included in the study.
